# Pectoral muscle removal in mammogram images: A novel approach for improved accuracy and efficiency

**DOI:** 10.1007/s10552-023-01781-0

**Published:** 2023-09-07

**Authors:** Simin Chen, Debbie L. Bennett, Graham A. Colditz, Shu Jiang

**Affiliations:** 1 Division of Public Health Sciences, Department of Surgery, Washington University School of Medicine, 660 South Euclid Avenue, St. Louis, MO MSC 8100-0094-02 USA; 2 Alvin J. Siteman Cancer Center, Barnes-Jewish Hospital and Washington University School of Medicine, St. Louis, MO USA; 3 Department of Radiology, Washington University School of Medicine, St. Louis, MO USA

**Keywords:** Breast evaluation, Full-field digital mammography, Pectoral muscle removal

## Abstract

**Purpose:**

Accurate pectoral muscle removal is critical in mammographic breast density estimation and many other computer-aided algorithms. We propose a novel approach to remove pectoral muscles form mediolateral oblique (MLO) view mammograms and compare accuracy and computational efficiency with existing method (Libra).

**Methods:**

A pectoral muscle identification pipeline was developed. The image is first binarized to enhance contrast and then the Canny algorithm was applied for edge detection. Robust interpolation is used to smooth out the pectoral muscle region. Accuracy and computational speed of pectoral muscle identification was assessed using 951 women (1,902 MLO mammograms) from the Joanne Knight Breast Health Cohort at Washington University School of Medicine.

**Results:**

Our proposed algorithm exhibits lower mean error of 12.22% in comparison to Libra’s estimated error of 20.44%. This 40% gain in accuracy was statistically significant (*p* < 0.001). The computational time for the proposed algorithm is 5.4 times faster when compared to Libra (5.1 s for proposed vs. 27.7 s for Libra per mammogram).

**Conclusion:**

We present a novel approach for pectoral muscle removal in mammogram images that demonstrates significant improvement in accuracy and efficiency compared to existing method. Our findings have important implications for the development of computer-aided systems and other automated tools in this field.

**Supplementary Information:**

The online version contains supplementary material available at 10.1007/s10552-023-01781-0.

## Introduction

Breast cancer is a leading cancer among women worldwide, accounting for 1 in 4 cancers diagnosed in women. The social and economic impact of this cancer underscores the importance of early detection and effective treatment. Mammography is widely used for breast cancer screening and typically involves acquiring two different views—the craniocaudal (CC) view and the mediolateral oblique (MLO) view. The CC view is obtained by imaging the breast in compression from a superior to inferior direction, while the MLO view is acquired from a mediolateral oblique angle (45°) which necessarily includes parts of the pectoral muscle from the chest that overlaps with the breast tissue. As we move to global use of digital mammography and increasingly need to integrate multiple exams over time to improve performance, efficient image processing and alignment are increasingly important [1].

Pectoral muscle removal, or segmentation, is a critical step in many computer-aided systems. In mammographic density estimation, for example, accurate removal of pectoral muscle is crucial in obtaining the correct dense tissue area/volume in relation to the total breast size. Automated diagnostic tools, on the other hand, also face challenges in analysis of breast tissue due to the presence of the pectoral muscle. This is particularly evident in the upper outer quadrant of the breast where the pectoral muscle can introduce increased noise, potentially interfering with the accuracy of image analysis. Thus, in the development of intricate pipelines for automated or computer-aided algorithm of breast tissue evaluation or cancer detection, the removal of the pectoral muscle is often considered a vital initial step that requires careful attention and prioritization.

In a recent study [2], comparison was made between two commonly used methods, namely Libra [3] and OpenBreast [4] for pectoral muscle removal in full-field digital mammogram (FFDM) images. That study included 168 women revealing that Libra exhibited superior performance in terms of accuracy when compared to OpenBreast. Our work, on the other hand, presents a novel approach that further improves the current methodology in pectoral muscle removal.

Through extensive evaluation on a large dataset of 951 women with 1,902 MLO-view mammograms, we demonstrate a superior accuracy in identifying and removing the pectoral muscle from FFDM mammogram images, along with improved overall efficiency in terms of computational time, when compared to Libra. Our findings offer a promising solution for enhanced image analysis in the context of breast tissue evaluation and mass detection, providing valuable insights for further advancements in the field.

## Method

### Study population

The Joanne Knight Breast Health Cohort (JKBHC) consists of over 10,000 women who undergo repeated mammography screening at Siteman Cancer Center and have been followed since 2010 [5]. All women in the cohort had a baseline mammogram at entry and completed a risk factor questionnaire. Full-field digital mammograms were obtained using the same technology (Hologic). Women with a history of cancer at baseline (except nonmelanoma skin cancer) were excluded from the cohort. Follow-up data until October 2023 were obtained through record linkages to electronic health records and pathology registries, as previously described [5]. Approximately 80% of participants had a medical center visit, including mammography and other health visits, within the past 2 years. All analyses performed in this study use the nested case–control cohort within JKBHC, where the pathology-confirmed breast cancer cases were matched to two controls sampled from the cohort based on month of mammogram and age at entry. After excluding women with breast implants and those with missing mammography images, we retained 294 cases and 657 controls. As pectoral muscle only appears in the mediolateral oblique (MLO) view full-field digital mammograms, we analyzed a total of 1,902 images.

### Pectoral muscle identification algorithm

All mammograms used in this analysis are for-presentation images of size 3328 × 2560 pixels and processed with Hologic. Our algorithm is written in both *Python* and *Matlab* and directly takes the for-presentation mammograms in the DICOM format without the need for any pre-processing. The proposed algorithm is also not restrictive to the size of the mammograms. We drew a 5% random sample of images to compare the internally drawn demarcation of pectoral muscle area against an expert radiologist (DLB) blinded to the pectoral muscle identification. The correlation between the two demarcation areas was *r* = 0.99.

**Fig. 1:**
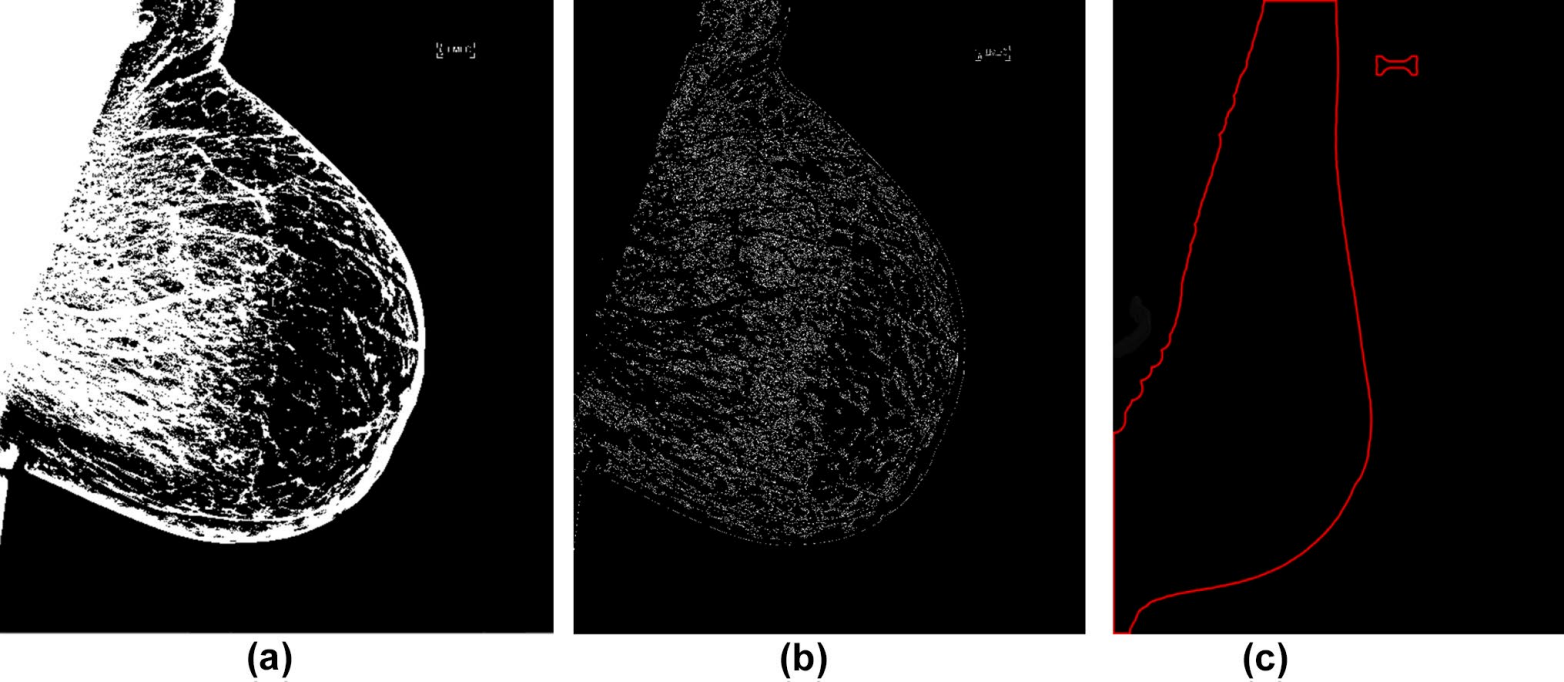
An example of the mammogram image **a** before; **b** after Canny algorithm for edge detection; and **c** with algorithm-detected edge of breast region

The proposed pectoral muscle identification pipeline is as follows. Initially, the image is subjected to binarization to enhance contrast with a global threshold. This process amplifies the distinction between highly bright pixels in the breast to less prominent ones [6]; see Fig. 1a as an example. Due to the presence of highly bright pixels in the pectoral muscle region, the enhanced contrast binarization procedure approximately delineates the breast tissue and the pectoral muscle. Following binarization, we applied the Canny algorithm [7] for the purpose of edge detection where a rough outer edge of the breast, excluding the pectoral muscle region, is found; see Fig. 1b. While there exist different ways for the purpose of edge detection, the Canny algorithm is very appealing for its simplicity and effectiveness in edge detection as discussed in several papers [8–10].

**Fig. 2:**
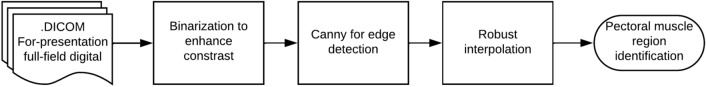
Flowchart for the pectoral muscle removal pipeline

Note that the detected edge of the breast is on the pixel level (Fig. 1b) and does not yet present a smooth edge. We thus propose to adopt a robust interpolation to smooth all the discontinuous regions presented within the mammogram [11]. As depicted in Fig. 1c, the periphery of the breast tissue is well estimated with the proposed algorithm. Because the algorithm automatically detects the breast tissue, the pectoral muscle, as a result, is consequently identified. We present the flowchart of our algorithm in Fig. 2.

### Statistical approach

**Fig. 3:**
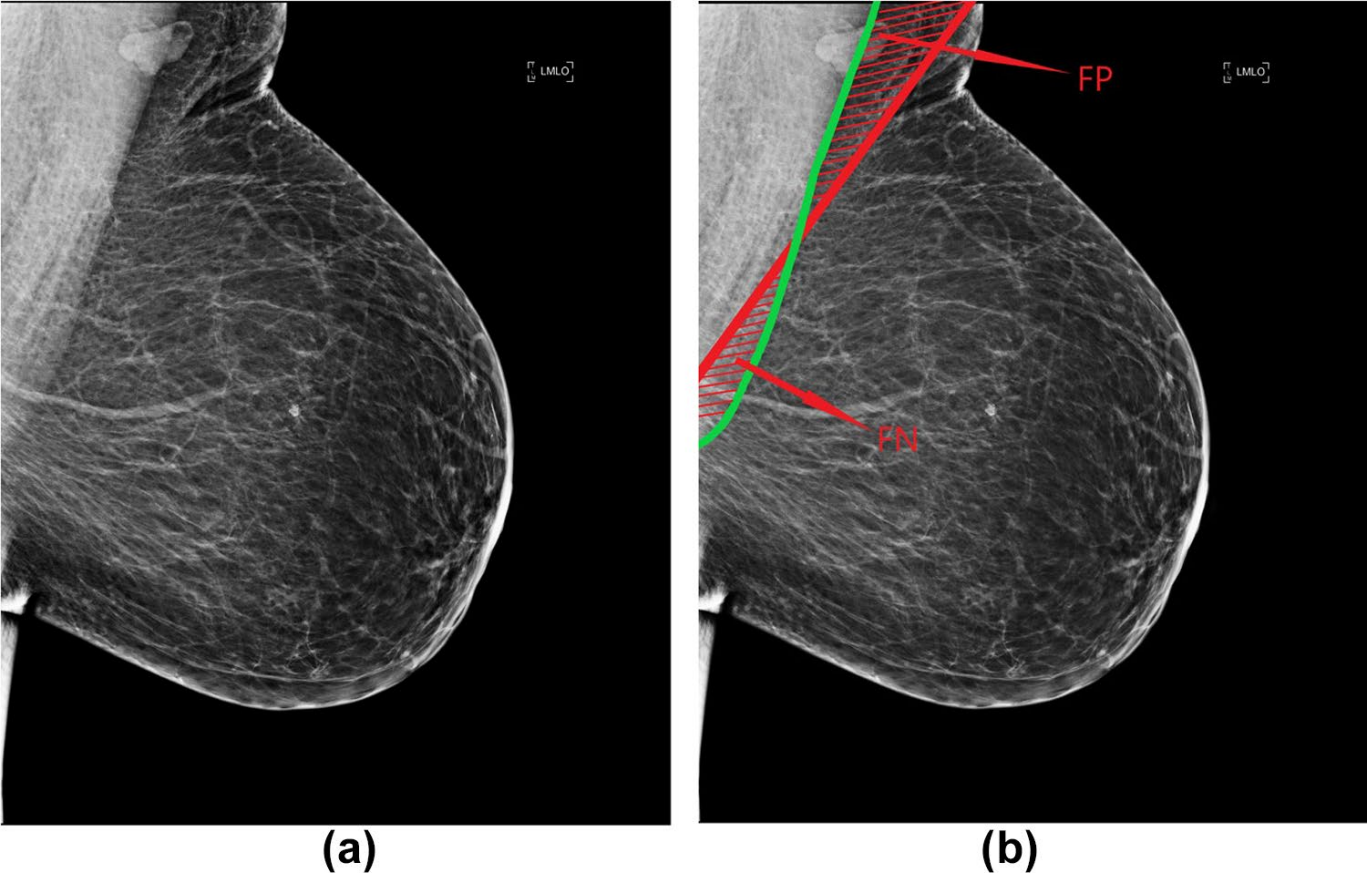
**a** The original mammogram. **b** The green line represents the true pectoral muscle region on the mammogram. The red line illustrates the false-positive regions (FP) and false-negative regions (FN)

We first demonstrate two distinct types of errors that can occur during the pectoral muscle identification progress, see Fig. 3. Specifically, with reference to the true pectoral muscle region, indicated by the green line, we define “false positives” (FP) as regions that are incorrectly identified as pectoral muscle despite being outside of the true region, and “false negatives” (FN) as regions within the true region that are erroneously identified as breast tissue.

We estimate the percentage of total pixels that make up the FP and FN with respect to the true pectoral muscle regions on each mammogram. Because prior findings identified Libra to be superior in terms of accuracy when compared to OpenBreast [2], we compare our proposed algorithm with Libra in the subsequent section. We present FP and FN for both the proposed method and for application of Libra to the same set of 1,902 study images. The two-sample Z test was used to test for the statistical significance between the proposed method and Libra. Additionally, we report the efficiency in terms of computational time for pectoral muscle removal in each MLO-view mammogram using the proposed method and Libra.

## Results

The risk factor profile for these women has been reported previously [1]. Women are Black (15%) white (81%) or other race/ethnicity. The mean age is 57 and 73% are postmenopausal.

**Fig. 4:**
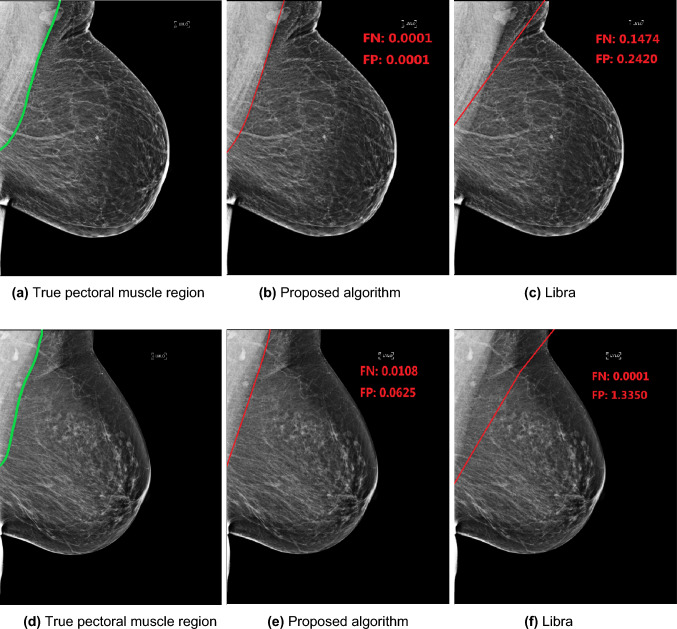
Two examples for pectoral muscle identification. The first column represents the true pectoral muscle region with comparison to regions identified using our proposed algorithm (second column) and Libra (third column)

For visualization purposes, we first show two examples in Fig. 4 where the first column represents the true pectoral muscles. We show the identified pectoral muscle region using our proposed algorithm (second column) in comparison to Libra (last column) with their corresponding FP and FN errors reported on each. In both examples, we can see that the pectoral muscle identified using the proposed algorithm is very close to the true region. Libra, on the other hand, tends to overestimate the pectoral muscle region by including areas that are within the breast.

The results from applying the proposed method and Libra over all 1,902 MLO mammograms are shown in Table 1. We see that on average, our proposed algorithm exhibits lower mean error of 12.22% in comparison to Libra’s estimated error of 20.44%. That is, the proposed algorithm minimizes 40% of the error compared to Libra when looking at the true positive and true negative regions together. This gain in accuracy was statistically significant (*p* < 0.001).

**Table 1:** The estimated false positive (FP) and false negative (FN) for both the left and right MLO views

		Proposed (%)	Libra (%)
Left MLO	FP	7.92	22.68
	FN	16.97	10.67
	Mean	12.45	17.18
Right MLO	FP	12.41	38.97
	FN	17.54	7.40
	Mean	13.98	23.69
Both	FP	9.17	30.83
	FN	17.23	9.04
	Mean	12.22	20.44

When separated out by type of error, FP and FN, we see that Libra tends to overestimate the FP by 30.83% compared to our proposed algorithm of 9.17%. On the other hand, our proposed algorithm tends to overestimate the FN by 17.23% compared to Libra of 9.04%.

When separated by sides, i.e., left and right, we see that Libra exhibits a 27.5% higher error rate for the right MLO mammograms compared to the left. However, the performance of proposed method remains mostly consistent. Interestingly, Supplementary Material Fig. S1 indicates that women have a relative larger pectoral muscle on the right side of their MLO mammograms; this difference in area is statistically significant using a two-sample t test (*p* < 0.001). This could be attributed to the fact that the majority of women are right handed.

The same set of results stratified by BMI > 25 and <  = 25 are also reported in Tables S1 and S2 within the Supplementary Material. We see that the proposed method outperforms Libra for both BMI > 25 (*n* = 583) and BMI <  = 25 (*n* = 368). The average error for FP and FN remains largely unchanged for the two strata when using the proposed method. However, Libra tends to have better performance (26.7% reduction in error) for those women with BMI <  = 25 in comparison to the stratum of BMI > 25.

**Fig. 5:**
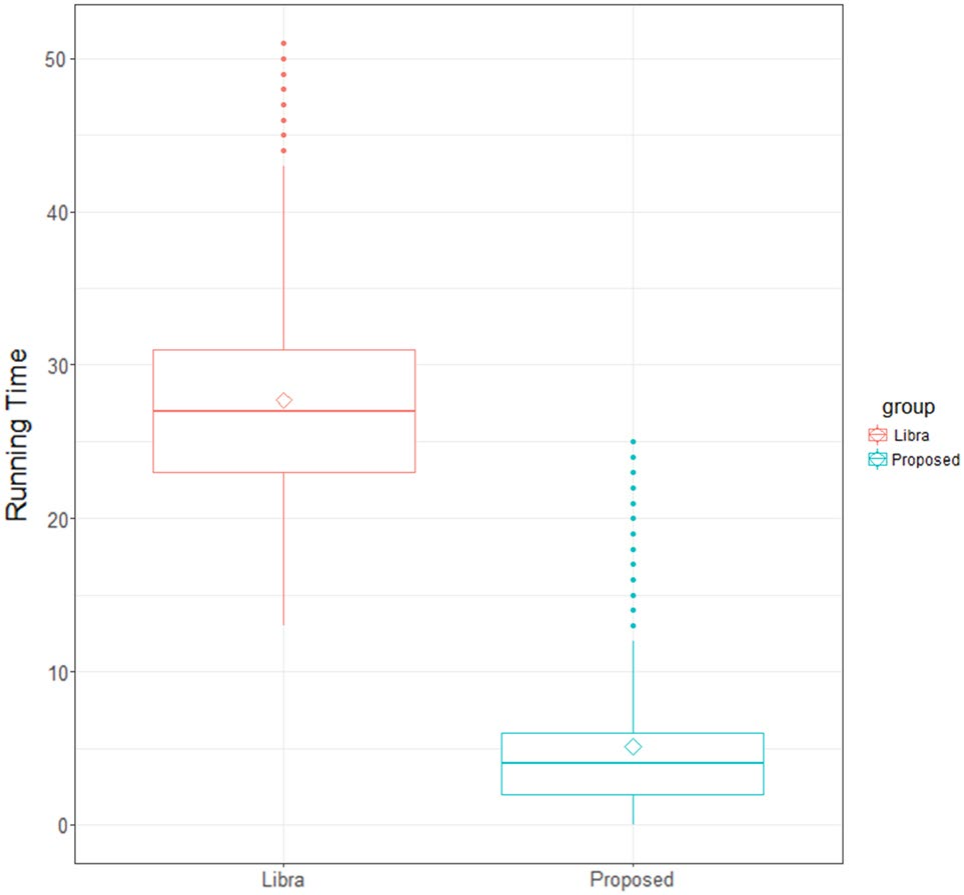
The distribution of computational/running time (in seconds) for pectoral muscle removal using the proposed method and Libra

Furthermore, our algorithm demonstrates significantly improved processing speed compared to Libra, see Fig. 5. When tested on the same dataset using the same laptop with no parallel computing, our algorithm takes, on average, 5.1 s (sd = 4.6) to output the pectoral muscle region per mammogram, whereas Libra takes approximately 27.7 s (sd = 6.0). This suggests an approximately 5.4 times efficiency gain in computational speed, which could significantly speed up future needs in pectoral muscle identification in other computer-aided algorithms.

## Discussion

Our study draws on routine screening mammograms from a prospective cohort and introduces a novel and efficient approach for pectoral muscle removal in full-field digital mammogram images that demonstrates improved accuracy and efficiency compared to Libra. The findings of our study have important implications for mammographic density estimation, computer-aided systems, and other automated tools used in breast cancer screening, diagnosis, and risk prediction. One of the key challenges in developing computer-aided systems in breast tissue evaluation and mass detection is the accurate removal of the pectoral muscle within MLO-view mammograms, which can interfere with the analysis of breast tissue.

Our extensive evaluation on a large dataset of 951 women with 1,902 MLO-view full-field digital mammogram images demonstrated the superior accuracy (*p* < 0.001) of our approach in identifying the pectoral muscle, thereby reducing risk of false-positive or false-negative muscle removal in subsequent image analysis. We demonstrated that our proposed method is robust across different breast compositions, including both fatty and dense breasts, as well as variation in the size of the pectoral muscle. Furthermore, our proposed approach also offers enhanced efficiency in terms of computational time (5.4 times more) compared to Libra. The reduced computational time is a significant advantage, as it can improve the overall performance of computer-aided systems by reducing processing time and increasing throughput, which is crucial for real-time or near-real-time applications in clinical settings.

Other studies have acknowledged the challenge of pectoral muscle removal. Studies of digitized screening film mammograms have manually removed pectoral muscle [12] and noted that consistency among different readers is not a straightforward task. Others have used computer programs to remove muscle from CC but not from MLO views [13, 14].

There are limitations to this study. First, our evaluation was based on full-field digital mammogram images, and further studies on datasets with digital breast tomosynthesis images may be needed. Second, our proposed approach has limitations in images with partially obscured or distorted pectoral muscle. While such constraint is also persistent in other existing methods, further research and refinement of the approach may be needed to address these limitations.

## Conclusion

Our study presents a novel approach for pectoral muscle removal in mammogram images that demonstrates 40% improved accuracy (*p* < 0.001) and 5.4 times more computationally efficient compared to Libra. Our findings contribute to the growing body of literature on image analysis for breast cancer screening and diagnosis and have important implications for the development of computer-aided systems and other automated tools in this field.

### Supplementary Information

Below is the link to the electronic supplementary material.

Supplementary file1 (DOCX 68 KB)

## Data Availability

All datasets were accessed and used under IRB approved protocols and are available from the corresponding author upon reasonable request.
