# Supplementary material for: Pectoral muscle removal in mammogram images: A novel approach for improved accuracy and efficiency
Source: Cancer Causes Control. 2023 Sep 7;35(1):185–91. doi: 10.1007/s10552-023-01781-0 (PMC10764470; doi:10.1007/s10552-023-01781-0)
Supplement: Supplementary file 1 — Supplementary file1 (DOCX 68 KB) [file 10552_2023_1781_MOESM1_ESM.docx]

**Supplementary Material for “Pectoral muscle removal in mammogram images: A novel approach for improved accuracy and efficiency.”**

Simin Chen, Debbie L. Bennett, Graham A. Colditz, Shu Jiang

**Figure S1.** The estimated area in pixels for true pectoral muscle regions on both the left and right MLO views.

^
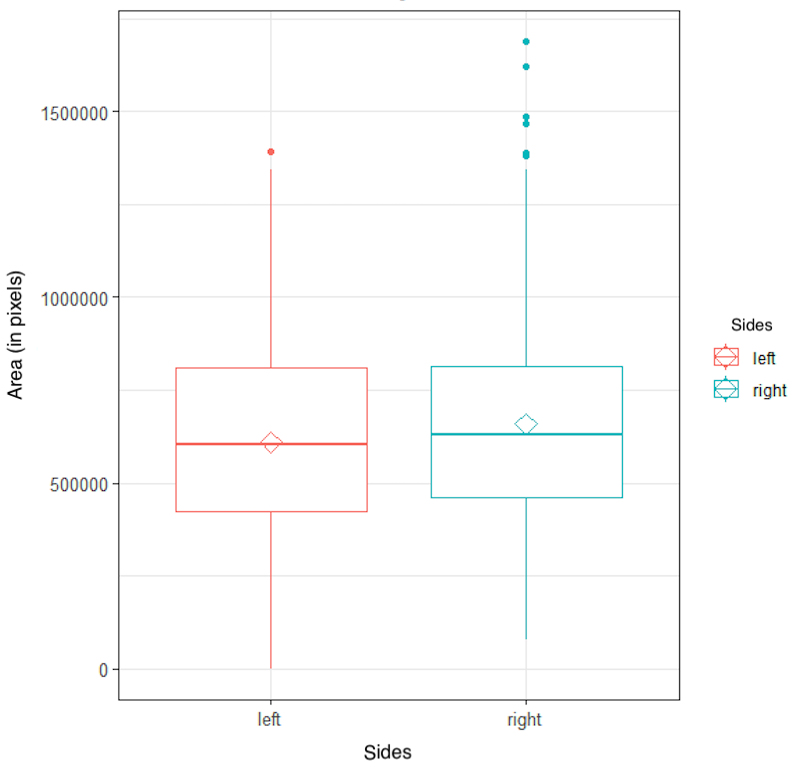
^

**Table S1.** The estimated false positive (FP) and false negative (FN) for both the left and right MLO views for those women with BMI > 25.

|  |  | **Proposed** | **Libra** |
| --- | --- | --- | --- |
| Left MLO | FP | 10.45% | 25.70% |
|  | FN | 12.16% | 10.05% |
|  | Mean | **11.31%** | **17.88%** |
| Right  MLO | FP | 11.19% | 41.90% |
|  | FN | 12.43% | 5.06% |
|  | Mean | **11.81%** | **23.48%** |
| Both | FP | 10.82% | 33.8% |
|  | FN | 12.30% | 8.81% |
|  | Mean | **11.56%** | **21.31%** |

**Table S2.** The estimated false positive (FP) and false negative (FN) for both the left and right MLO views for those women with BMI <= 25.

|  |  | **Proposed** | **Libra** |
| --- | --- | --- | --- |
| Left MLO | FP | 3.93% | 17.91% |
|  | FN | 24.56% | 10.64% |
|  | Mean | **14.25%** | **14.28%** |
| Right  MLO | FP | 3.92% | 29.00% |
|  | FN | 20.54% | 4.95% |
|  | Mean | **12.23%** | **16.98%** |
| Both | FP | 3.93% | 23.46% |
|  | FN | 22.55% | 7.80% |
|  | Mean | **13.24%** | **15.63%** |
